# Nickel-Based High-Bandwidth Nanostructured Metamaterial Absorber for Visible and Infrared Spectrum

**DOI:** 10.3390/nano12193356

**Published:** 2022-09-27

**Authors:** Rana Muhammad Hasan Bilal, Muhammad Ahsan Saeed, Muhammad Ashar Naveed, Muhammad Zubair, Muhammad Qasim Mehmood, Yehia Massoud

**Affiliations:** 1 Innovative Technologies Laboratories (ITL), King Abdullah University of Science and Technology (KAUST), Thuwal 23955, Saudi Arabia; 2 School of Electrical Engineering, Korea University, Seoul 02841, Korea

**Keywords:** metamaterial, absorber, nickel, nanostructured, polarization insensitive, visible, high bandwidth

## Abstract

The efficient control of optical light at the nanoscale level attracts marvelous applications, including thermal imaging, energy harvesting, thermal photovoltaics, etc. These applications demand a high-bandwidth, thermally robust, angularly stable, and miniaturized absorber, which is a key challenge to be addressed. So, in this study, the simple and cost-effective solution to attain a high-bandwidth nanostructured absorber is demonstrated. The designed nanoscale absorber is composed of a simple and plain circular ring of nickel metal, which possesses many interesting features, including a miniaturized geometry, easily fabricable design, large operational bandwidth, and polarization insensitivity, over the previously presented absorbers. The proposed nanoscale absorber manifests an average absorption of 93% over a broad optical window from 400 to 2800 nm. Moreover, the detailed analysis of the absorption characteristics is also performed by exciting the optical light’s various incident and polarization angles. From the examined outcome, it is concluded that the nanostructured absorber maintains its average absorption of 80% at oblique incident angles in a broad wavelength range from 400 to 2800 nm. Owing to its appealing functionalities, such as the large bandwidth, simple geometry, low cost, polarization insensitivity, and thermal robustness of the constituting metal, nickel (Ni), this nano-absorber is made as an alternative for the applications of energy harvesting, thermal photovoltaics, and emission.

## 1. Introduction

In recent years, nanostructured metamaterials (NMMs), also called artificial composite nano-resonators, have received immense research interest in the research community because of their distinctive electromagnetic properties, namely anomalous refraction, optical cloaking, multiplexed holograms, polarization transformation, bio-chemical sensing, intelligent reflective devices, and many more [1,2,3,4,5,6,7,8,9,10,11,12]. The electromagnetic properties of the artificially engineered NMMs are strongly influenced by the structural parameters or constituent materials instead of their composition [13,14,15,16]. Since their first conceptualization in 1968 by Veselago et al., NMMs have been utilized in a wide range of engineering applications, including chiral imaging, super lenses, perfect absorbers, color filtering, and thermal imaging [17,18,19,20,21,22,23,24,25,26,27,28]. Therein, the metamaterial absorber (MMA) was firstly proposed by Landy et al. in 2008 [29], and thereafter, plenty of device architectures have been designed for microwave, terahertz, and optical frequencies [15,30,31,32,33,34,35,36,37]. Thenceforth, various functionalities, including wideband, narrow-band, polarization sensitive or insensitive, etc., have also been established in these MMAs [19,20,38,39,40].

Generally, in MMAs, surface plasmon polaritons, microcavity resonance, and electromagnetic polaritons based on strong light–matter interactions are used to secure an improved optical absorption in a wide or narrow wavelength zone [41,42]. Narrow-band absorbers are usually employed in applications centered on filtering or optical sensing [16,43,44,45]. However, some areas, such as thermal emitters, stealth technology, or photovoltaic applications, are highly required for perfect absorption over a wide range of wavelengths [46,47,48]. Wideband absorbers are preferred over narrow-band for such applications. Hence, various strategies have been devised to obtain wideband optical absorption, such as multiple resonators, a multilayer structure, and metallic gratings [20,49]. However, these strategies mostly involve complex fabrication processes, high material costs, and multifaceted designs that restrict their long-term practical applications. Therefore, researchers have explored diverse strategies to secure wideband absorption characteristics.

For example, Qi et al. obtained wideband absorption by designing a perfect cylindrical-based MMA. They achieved approximately 90% absorptivity covering a wide wavelength range from the ultraviolet (UV) to infrared region [50]. Similarly, Xie et al. developed a multi-band MMA that could generate up to four narrow-band peaks with a rectangular resonator [51]. Moreover, an average absorption of over 91% in the visible to the infrared region has been achieved in a multiscale geometry comprised of varied metallic nanoscale gaps. In another study, Zhou et al. developed close-packed resonator clusters in the nanotubes to widen the absorption bandwidth. In addition, they fine-tuned the diameters of nanoporous templates to provide variable resonance frequencies [52]. Likewise, Hoa et al. achieved absorption of over 90% in a wide range of 480 to 1480 nm with a multilayer nano-absorber comprising a top meta-structure of 10 metal/dielectric layers [53]. Nonetheless, these strategies have the disadvantages of a relatively low bandwidth, a multifaceted cell pattern, and the involvement of noble metals (e.g., gold, silver, aluminum, etc.). It is believed that the MA absorption can be enhanced by judiciously choosing the unit cell design, structural parameters, and the right choice of metal.

Noble metals are costly and show poor thermal stability due to low melting points that limit their utility in a high-temperature processing environment [20]. On the contrary, various metallic materials such as chromium (Cr), nickel (Ni), tungsten (W), and the nitrides of various refractory metals, including titanium nitride (TiN), zirconium nitride (ZrN), vanadium nitride (VN), and hafnium nitride (HfN), have shown promising potential as a unit cell element in MMAs because of their superior characteristics that include a low cost, excellent chemical stability, and a high melting point [21,33,54,55,56]. Likewise, the bulky size of photonic devices and the low bandwidth can be resolved by employing fractals that are repeated geometric structures arranged in a specific pattern. Based on repeated patterns, fractals produce multi-resonant characteristics corresponding to wideband absorption peaks in a single unit cell [57]. Our recent study [21] developed a wideband MMA based on a novel hexagonal nano-ring-shaped fractal geometry. The proposed fractal MMA demonstrated an ultra-compact thickness, angular robustness, and absorptivity of over 97% in a wide wavelength region (820–2520 nm). However, fractals based on a hexagonal structure increase the fabrication complexity which may limit their practicality. Therefore, it is still a quite challenging task to design a perfect broadband absorber having the simultaneous characteristics of simplicity, cost-effectiveness, and high absorption in the wide wavelength range.

In the present communication, the author’s motivation is to implement the simple and cost-efficient design architecture of the nano-absorber, which showed ultrabroad bandwidth, polarization insensitivity, single-layer device configuration, and thermal endurability. The nano-absorber has two metallic layers, and the middle layer is sandwiched between the two metallic layers. The top and bottom layers are made of nickel metal; a dielectric layer is a SiO_2_ substrate. The nickel (Ni) is integrated owing to its high melting point, corrosion resistance, and low price as compared to the noble metals, namely gold (Au), silver (Ag), etc. The proposed nano-absorber exhibits an average absorption of more than 93% on a large wavelength spectrum from 400 to 2800 nm. Moreover, the incident and polarization angles analysis on absorption characteristics are also characterized to notice its angular stability. In light of the above features, this nano-absorber could be useful for solar harvesting, thermal emission, and other optoelectronic applications.

## 2. Design Architecture and Simulation of Unit Cell 

**Figure 1:**
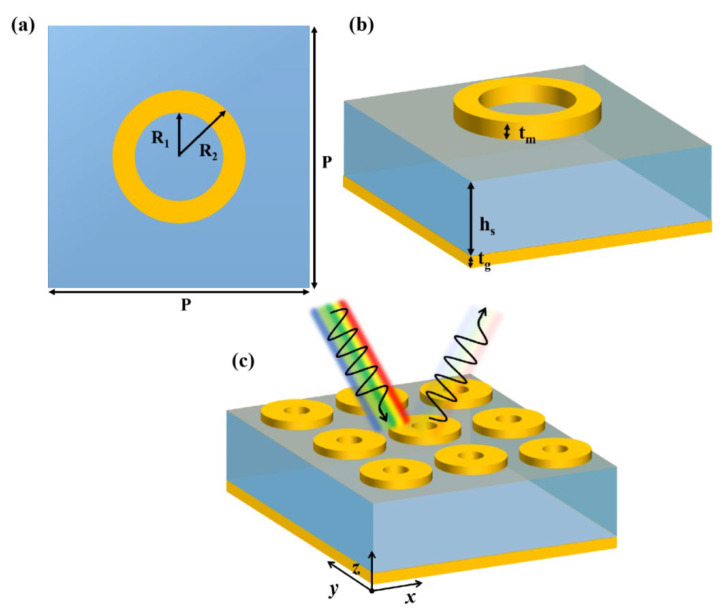
Schematic depiction of the proposed nano-absorber: (**a**) front outlook of the proposed meta-cell composed of nano circular ring of nickel (Ni), (**b**) side view of the proposed unit cell of the nano circular ring of nickel (Ni), and (**c**) 3D periodic array of the unit cells of the of nano circular ring of nickel (Ni).

The unit cell schematic of the proposed nano metamaterial absorber (NMMA) is depicted in Figure 1. The unit structure is comprised of three layers. The top and bottom layers are composed of nickel, and quartz (SiO_2_) is used as a substrate sandwiched between them. The electrical conductivity of nickel is σ = 1.44 × 10^7^ S/m. The dielectric constant and loss tangent of quartz (SiO_2_) is equal to ε_r_ = 3.75 and tanδ = 0.0004, respectively [21]. The top layer is designed so that incidence waves cannot reflect and control the electromagnetic waves. The top layer of nickel is a simple and planar circular ring integrated on a dielectric substrate. It is simple and easily fabricable through modern photolithography and nanoimprint lithography techniques [58,59]. In the literature, numerous wideband nanostructured MMAs are reported which have complex and multifaceted design in comparison with our proposed NMMA. The contributing nano circular ring provides the extra degree of freedom to fabricate the prototype of this absorber due to its planar geometry and design simplification.

Furthermore, it is stable for broadband incidence angle because it is symmetrical from all directions. Oblique incidence angular stability mainly depends on the symmetry of structures. If the unit cell structure contains asymmetry at some portion, then results will be affected by the variation of incidence angle. The optimal structure of the unit cell is shown in Figure 1. The thickness of the substrate is denoted as h_s_, which is equal to 60 nm. The thickness of the top and bottom layers is denoted by t_m_ and t_g_, which are equal to 15 and 50 nm, respectively. The outer and inner radii of the nickel ring are equal to R_1_ = 60 nm and R_2_ = 80 nm. The periodicity of the unit cell is denoted by P, which is equal to 200 nm. For simulation, CST-2018 software was used. A frequency-domain solver is deployed to calculate the absorption numerically.

**Figure 2:**
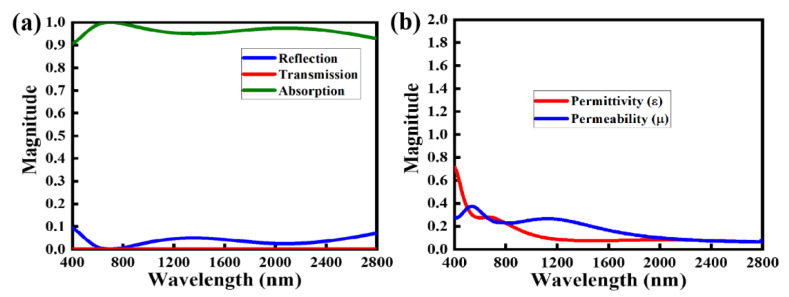
Optical characteristics of the proposed NMMA: (**a**) scattering parameters of the proposed NMMA, solid green line denotes the absorption curve, whereas red and blue solid lines represent the transmission and reflection parameters of the proposed NMMA, respectively, and (**b**) absolute value of the permittivity and permeability of the proposed NMMA.

The incident electromagnetic wave is propagating along the negative z-axis. That is why the “open add space” boundary condition is employed along Z_min_ and Z_max_. The same unit cell structure is repeatedly spread along the x-axis and y-axis, so the “unit cell” option is specified for boundary conditions. We define TE and TM modes as electric fields or magnetic fields transverse along the y-axis. Figure 2a,b depict our study’s simulation results, using TM and TE modes. The electromagnetic wave is intruded on the meta-absorber from the z-axis, and it enters the structure because of the impedance matching between free space and the meta-absorber. As the wave strikes any surface, there will be a reflection R(λ), transmission T(λ), and absorption A(λ) which can be related as $A\left(\lambda \right)=1-R\left(\lambda \right)-T\left(\lambda \right)$. The meta-absorber structure consists of the top layer (ring geometry), the substrate quartz (SiO_2_), and the bottom metallic layer (nickel), so the transmission is zero (T(λ) = 0), and absorption can be estimated through the reflection of wave meta-absorber ($A\left(\lambda \right)=1-R\left(\lambda \right))$. Total reflection can be calculated through $R\left(\lambda \right)={\left|S_{11}\right|}^{2}$ as $S_{11}$ corresponds to total reflected energy [33].

## 3. Results and Discussion

Achieving suitable optical characteristics of absorbers is essential to maximize the NMMA’s performance. Figure 2a depicts the optical properties, including the absorption, reflection, and transmission of the proposed NMMA. The NMMA demonstrates the average absorption of over 93% over a broad range of wavelengths (400–2800 nm) with a maximum absorption value at λmax = 650 nm, while the corresponding reflection and transmission spectra were reduced to the lowest level during the same wavelength span. The absorption profiles of the NMMA were obtained considering incident waves at $\theta =0^{{}^{\circ}}$, normal to the NMMA.

Further, the reflection coefficient for the normally incident optical light can be analyzed using the following equation [20].

(1) $$\Gamma \left(\omega \right)=\frac{Z_{M}-Z_{o}}{Z_{M}+Z_{o}}$$

The impedance of the nano-absorber is symbolized as $Z_{M}$ and it depends on the frequency, whereas the impedance of the free space is given as $Z_{o}$. The following mathematical expression gives these impedances:

(2) $$Z_{M}=\sqrt{\frac{\mu_{M}\mu_{o}}{\varepsilon_{M}\varepsilon_{o}}}$$

(3) $$Z_{o}=\sqrt{\frac{\mu_{o}}{\varepsilon_{o}}}=377\Omega$$

In Equations (2) and (3), $\varepsilon_{o}$ and $\mu_{o}$ signify the permittivity and permeability of the free space while $\varepsilon_{M}$ and $\mu_{M}$ characterize the effective permittivity and permeability of the nano-absorber, respectively. The permittivity and permeability of the nano-absorber are associated with the reflection and transmission coefficients as follows [60].

(4) $$\varepsilon_{M}=\frac{2}{\sqrt{-kd}}\frac{1-\left(S_{21}+S_{11}\right)}{1+\left(S_{21}+S_{11}\right)}$$

(5) $$\mu_{M}=\frac{2}{\sqrt{-kd}}\frac{1-\left(S_{21}-S_{11}\right)}{1+\left(S_{21}-S_{11}\right)}$$

where d is the height of the substrate and k shows the wavenumber and k=ω/c. As $S_{11}$ and $S_{21}$ go to zero, $\varepsilon_{M}$ and $\mu_{M}$ become equal, and $Z_{M}$ from Equation (7) is matched to $Z_{o}$. The impedance of the nano-absorber depends on the relative permittivity ($\varepsilon_{r}$) and relative permeability ($\mu_{r}$). As stated earlier, the impedance of the nano-absorber is given by ZM=μMμoεMεo (where $\varepsilon_{M}$ and $\mu_{M}$ show the permittivity and permeability of the nano-absorber, respectively). If the relative permittivity and relative permeability of the meta-structure are equivalent, the normalized impedance $Z/Z_{o}$ of the nano-absorber is unity which satisfies the matching condition for perfect absorption. For the discussed nano-absorber, the absolute values of permittivity and permeability are identical to each other in a large optical window (400–2800 nm), as shown in Figure 2b. So, it is obvious from Figure 2a that the absorption of the nano-absorber is over 90% for the above-mentioned optical window, i.e., 400–2800 nm.

In order to completely understand the proposed nano-absorber, the key material parameters, namely the relative permittivity, relative permeability, impedance, and refractive index, are plotted using S-parameters. When the S-parameters are gathered by simulating the nano-absorbing device, these parameters can be computed using the following equations [61,62].

(6) $$n=\frac{-iln\left(e^{ink_{0}d}\right)}{k_{0}d}$$

(7) $$e^{ink_{0}d}=X\pm i\sqrt{1-X^{2}}$$

(8) $$=\frac{1}{2S_{21}\left(1-S_{11}^{2}+S_{21}^{2}\right)}$$

In the above series of Equations (6)–(8), $k_{0}$ and d denote the wavenumber and thickness of the absorber, respectively, and n is the refractive index of the nano-absorber. The relative permittivity, $\varepsilon_{r}$, and relative permeability, $\mu_{r}$, can be computed as follows [61,62].

(9) $$\varepsilon_{r}=\frac{n}{Z}$$

(10) $$\mu_{r}=nZ$$

**Figure 3:**
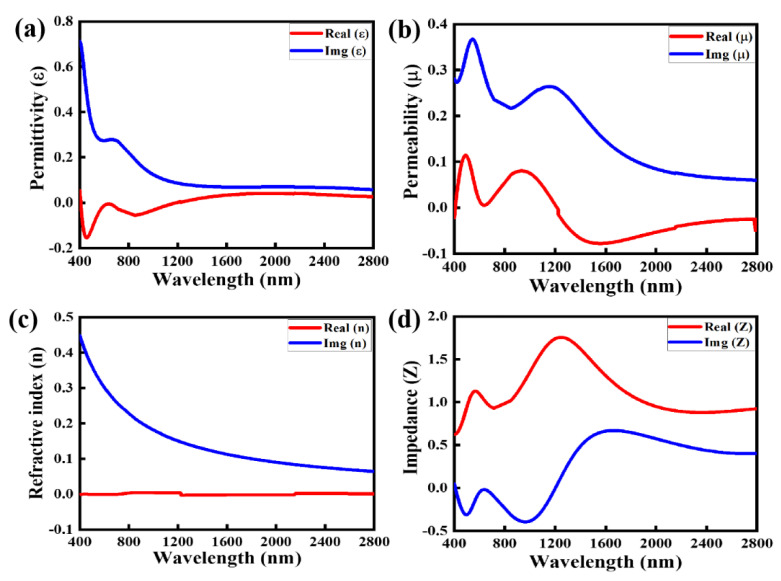
Optical material parameters of the proposed NMMA: (**a**) complex value of permittivity (ε), (**b**) complex value of permeability (µ), (**c**) complex value of refractive index (n), and (**d**) complex value of the impedance (Z).

**Figure 4:**
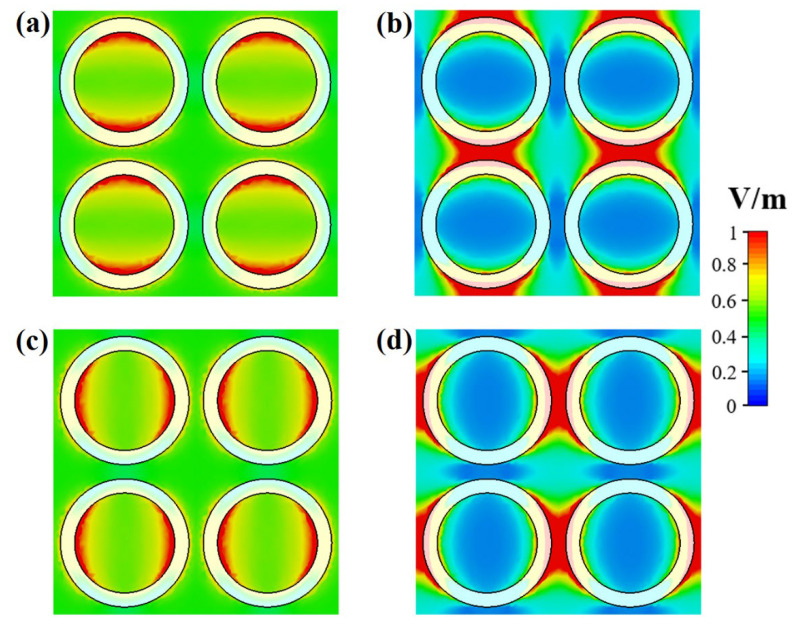
Surface electric field distribution of the proposed NMMA: (**a**) TE-wave polarized at λ = 650 nm, (**b**) TE-wave polarized at λ = 2550 nm, (**c**) TM-wave polarized at λ = 650 nm, (**d**) TM-wave polarized at λ = 2550 nm.

Figure 3a,b illustrate the real and imaginary parts of the relative permittivity ($\varepsilon_{r}$) and relative permeability ($\mu_{r}$), respectively, and Figure 3c illustrates the complex values of the refractive index. Because the $\varepsilon_{r}$ and $\mu_{r}$ depend on the S-parameters, the equation gives the normalized effective impedance of the surface. Figure 4d demonstrates that the real value of the normalized complex impedance is approaching toward 1 and the imaginary part is fluctuating between 0 and 0.5 over a large optical window, which confirms the impedance match between the meta-structure and free space. Therefore, the proposed NMMA exhibits an average absorption rate of over 93% from 400 to 2800 nm. The normalized effective impedance of the proposed NMMA is given by the following equation [20].

(11) $$Z_{eff}=\sqrt{\frac{{\left(1+S_{11}\right)}^{2}-S_{21}^{2}}{{\left(1-S_{11}\right)}^{2}-S_{21}^{2}}}=\frac{1+S_{11}}{1-S_{11}}$$

Figure 4a,b show the surface electric field of the designed nickel-based nano-absorber for two different operating wavelength points (λ = 650 and 2550 nm) at which it induces two plasmonic resonances. The top row of Figure 4 explicates the electric field accumulation of the TE-wave polarized light of the electromagnetic spectrum. It is visibly observed that the electric field is maximally confined to the top-inner and bottom-inner portion of the nickel-based circular unit cell. The magnitude of the electric field is prominently increased toward the outer side of the circular ring at the top and bottom portion for the operating wavelength of 2550 nm. Further, the dipole-like phenomena are generated in Figure 4 at two wavelengths, where the plasmonic resonances are generated. Moreover, the bottom row of Figure 4 highlights the electric field characteristics of the proposed nano-absorber by considering the TM-wave polarized light. In fact, the TE- and TM-modes are perpendicular to each other, so their electric field distribution is also perpendicular to each other, as clearly noticed in Figure 4. Resultantly, these electric field distributions induce the electric resonance that causes the absorption.

**Figure 5:**
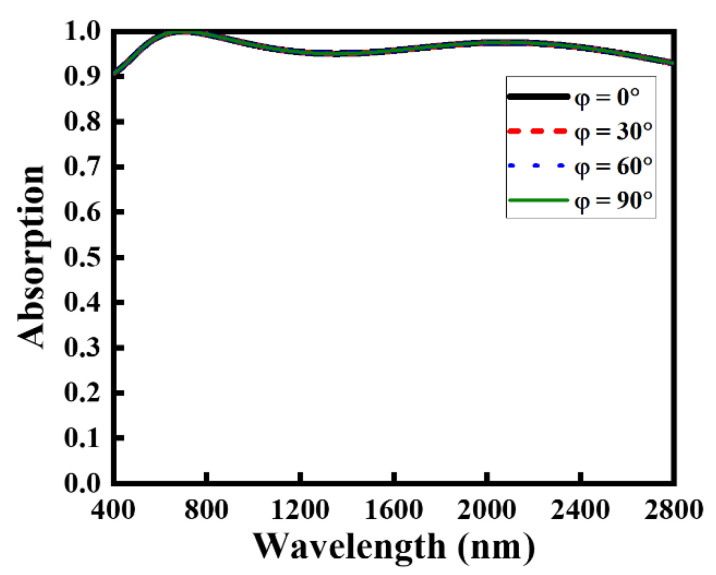
The optical absorption rate of the proposed NMMA under the variation of polarization angles of the incoming light.

Next, we examine the dependence of the absorption on the polarization angles to evaluate the angular stability of the proposed absorber configuration. Figure 5 shows the absorption profile of the NMMA under various polarization angles ranging from 10° to 90°. It was observed that the NMMA exhibits a uniform and stable absorption profile at all indicated angles (0–90°) that can be attributed to the symmetric nature of the participating circular ring [63,64,65]. Because the shape of the proposed absorber has a four-fold symmetry in its design, it shows a polarization-insensitive response (the absorption is identical for all the polarization angles by considering the normal incident case, $\theta =0^{{}^{\circ}}$).

The reflection coefficients are strongly influenced by the oblique incident and refraction angles [20,21].

(12) $$\Gamma_{TE}=Z_{m}cos\theta_{i}-Z_{o}cos\theta_{t}/Z_{m}cos\theta_{i}+Z_{o}cos\theta_{t}$$

(13) $$\Gamma_{TM}=Z_{m}cos\theta_{t}-Z_{o}cos\theta_{i}/Z_{m}cos\theta_{t}+Z_{o}cos\theta_{i}$$

where $Z_{m}$ is the impedance of the metamaterial, $Z_{o}$ represents the impedance of the free space, and $\theta_{i}$ and $\theta_{t}$ denote the incidence and transmitting angle, respectively. Henceforth, by Snell’s law,

(14) $$\frac{Z_{o}}{Z_{m}}=\frac{sin\theta_{t}}{sin\theta_{i}}$$

From Equations (1) and (2), the maximum achieved absorption for the TE- and TM-polarization can be written as

(15) $$\left(A_{TE}\right)=\mu \varepsilon -\varepsilon^{2}sin^{2}\theta_{i}-\mu^{2}cos^{2}\theta_{i}=0,$$

(16) $$\left(A_{TM}\right)=\mu -\varepsilon sin^{2}\theta_{i}-\mu \varepsilon cos^{2}\theta_{i}=0.$$

**Figure 6:**
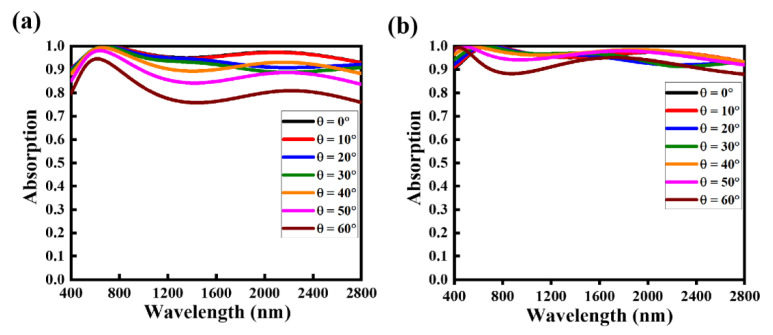
Optical absorption rate of the proposed NMMA under the variation of incident angles of the incoming light: (**a**) TE-wave polarized optical light and (**b**) TM-wave polarized optical light.

Further, the absorption profiles of the NMMA for both polarizations (TE and TM) are shown in Figure 6a,b. At lower oblique incidence angles (*θ* < 30°), both the TE and TM modes exhibit relatively high absorption of over 85% with some fluctuations. For the TE mode, the NMMA maintains a high absorption efficiency of over 80% up to *θ* = 50° in a wide wavelength range (300–2800 nm) as depicted by the pink line. By contrast, a better absorption profile of over 85% was achieved in the TM mode up to *θ* = 60° in the same wavelength region, with a near-unity absorption in the visible wavelength region. Overall, the proposed NMMA exhibits excellent absorption characteristics (80%) at varied oblique incident angles in a broad wavelength range, demonstrating its robustness. In each incident angle, the incoming optical light has different rotations, and the proposed design faces an anisotropic effect causing the variation in absorption.

Table 1 helps to understand the comparative analysis of this research with the previously published work on MMAs. In the present study, we used a single nano-ring of Ni which has many advantages over the reported studies, including a simple geometry, being easily fabricable, large operational bandwidth, etc., whereas the remaining design architectures are either composed of multiple-stacked layers or have a lower operational bandwidth as compared to our proposed NMMA. So, in light of all the mentioned attributes, our NMMA would be a great addition in the stream of nanostructured absorbers.

**Figure 7:**
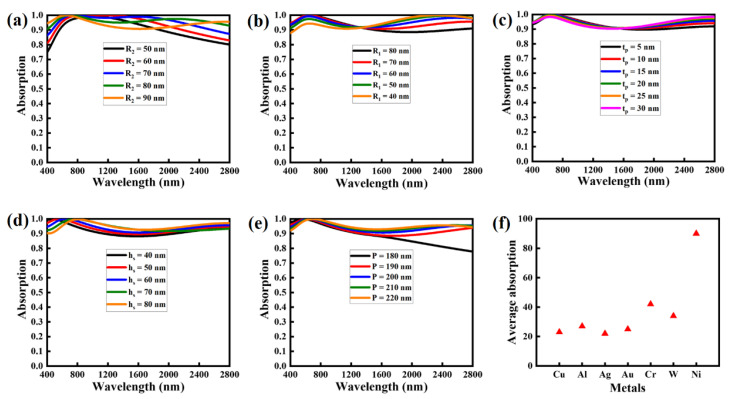
Optical absorption rate of the proposed NMMA under the variation of various geometric parameters: (**a**) outer radius of the top nano circular ring, (**b**) inner radius of the top nano circular ring, (**c**) thickness of the top nano circular ring, (**d**) thickness of the dielectric spacer, (**e**) periodicity of the unit cell, and (**f**) comparison of the average absorption rate of the different metals, which are used in designing the top nano circular ring.

Further, the effects on the absorption properties of the major structural parametric variation are shown in Figure 7. The outer radius of the nickel ring is varied in the range of 50 to 90 nm, as shown in Figure 7a. The averaged absorption of the NMMA remains near unity at radii of 60 and 70 nm in the wavelength range of 450 to 1600 nm. In addition, at the radius of 80 nm, the overall averaged absorption remains stable around 90% during the entire wavelength range. Meanwhile, at the higher radius (90 nm), the absorption tends to decrease in the mid-wavelength region (800–1200 nm), followed by increasing behavior at the higher wavelength region. Overall, the NMMA demonstrates an averaged absorption of over 90% at all radii for the entire wavelength region (400–2800 nm). Thereafter, the inner radius of the nickel ring is also altered from 40 to 80 nm to highlight its impact on the absorption features of the proposed NMMA. Figure 7b displays the absorption results and it is obvious in Figure 7b that it remains above 90% for all the mentioned values (R_1_ = 40–80 nm), but it slightly decreases at the higher value of R_1_. 

Furthermore, the impact of the thickness of the nickel nano-ring is also inspected by varying its value from 5 to 30 nm. It is noticed in Figure 7c that the t_p_ has almost no significant contribution to change the absorption characteristics of the proposed NMMA. Next, the dielectric substrate’s thickness varies between 40 and 80 nm to examine the thickness-dependent absorption evolution of the NMMA (Figure 7d). At shorter wavelengths (<800 nm), the NMMA demonstrates stable absorption and is not strongly influenced by the substrate thickness variation. In the mid-wavelength region (1000–2000 nm), a decrease in the thickness of the substrate results in the decline of the NMMA’s absorption with an average value of approximately 90%. At wavelengths >2000 nm, the NMMA exhibits an increase in the absorption with the maximum value of 95% at h_s_ = 80 nm. 

The impact of the variation in the periodicity of the unit cell on the absorption of the NMMA is shown in Figure 7e. By decreasing the P of the unit cell, an increment in the absorption of the NMMA is observed, with averaged values of around 90%. Though, at the lowest P (180 nm), the absorption was substantially decreased after 2000 nm, yielding the minimum value of around 70% at the longer wavelength. Overall, the variation in the P has not significantly influenced the NMMA’s absorption characteristics. 

For comparison, the averaged absorption of the absorber comprising various metals, including Cu, Al, Ag, Au, Cr, W, and Ni, is illustrated in Figure 7f. It is evident that Ni has the highest averaged absorption of over 93%, almost three-folds higher than all other metals with the same structural parameters in all cases. The metals Cu, Al, Ag, and Au exhibited an averaged absorption below 30%, while Cr and W showed an absorption of 42 and 35%, respectively. 

## 4. Conclusions

This paper investigated a simple and easily manufactured large bandwidth visible-infrared Ni-based nano-absorber for a large wavelength window. The discussed nano-absorber comprises a planar circular ring of Ni metal grown over a lossy dielectric substrate of SiO_2_, which is backed by a Ni-plated ground sheet. The proposed absorber indicates an average absorption value of 93% for a broad optical window, starting from 400 to 2800 nm. In addition, the absorption features were also inspected under the rotation of incident and polarization angles of the incoming optical light. It was found that the absorber maintained its average absorption of 80% till *θ* = 60° and also noted the polarization-insensitive behavior is owing to the symmetric geometry of the Ni-based circular ring. Furthermore, the absorption characteristics were also analyzed under the influence of different design parameters of the nano-absorber. The surface electric field plots were also studied to highlight the absorption mechanism. Finally, this type of nanostructured absorber could find interesting applications in thermal imaging, energy harvesting, and thermal photovoltaics.

## Figures and Tables

**Table 1:** Comparison of the reported metamaterial-based nano-absorbers with the present study.

Design Configuration	Material	Device Layers	Optical Bandwidth (A ≥ 90%)	Angular Robustness	Polarization Insensitivity
Stacked-layers [20]	TiN, TiO_2_	Multiple	200–2800 nm	θ = 60°	Yes
Multiple-hexagons (Fractal) [21]	SiO_2_, Ni	Single	820–2700 nm	θ = 60°	No
Nano-ellipses (Fractal) [33]	SiO_2_, W	Single	400–750 nm	θ = 60°	No
Nano-cylinders [52]	Ni	Single	400–650 nm	θ = 60°	Yes
Frustum-like nano-cones [53]	Si, Au	Multiple	480–1480 nm	θ = 60°	Yes
Nano-triangular rings [54]	SiO_2_, Cr	Single	400–750 nm	θ = 60°	Yes
Nano-pillars [66]	Ni	Single	400–760 nm	θ = 70°	Yes
Nano-cubes [67]	SiO_2_, Ti, MgF_2_, Al	Multiple	400–1500 nm	θ = 60°	Yes
Nano-disks [68]	SiO_2_, TiN, TiO_2_	Multiple	316–1426 nm	θ = 50°	Yes
Nano-hexagonal rings [69]	AlN, Ni	Single	380–2500 nm	θ = 60°	Yes
Nano-circular ring (This study)	SiO_2_, Ni	Single	400–2800 nm	θ = 60°	Yes

## Data Availability

All the relevant data are presented in this research article but may be obtained from the authors upon reasonable request.
